# Attention-based approach to predict drug–target interactions across seven target superfamilies

**DOI:** 10.1093/bioinformatics/btae496

**Published:** 2024-08-08

**Authors:** Aron Schulman, Juho Rousu, Tero Aittokallio, Ziaurrehman Tanoli

**Affiliations:** Institute for Molecular Medicine Finland (FIMM), HiLIFE, University of Helsinki, Helsinki, 00014, Finland; Department of Computer Science, Aalto University, Espoo, 02150, Finland; Institute for Molecular Medicine Finland (FIMM), HiLIFE, University of Helsinki, Helsinki, 00014, Finland; iCAN Digital Precision Cancer Medicine Flagship, University of Helsinki and Helsinki University Hospital, Helsinki, 00014, Finland; Department of Cancer Genetics, Institute for Cancer Research, Oslo University Hospital, Oslo, 0379, Norway; Oslo Centre for Biostatistics and Epidemiology (OCBE), Faculty of Medicine, University of Oslo, Oslo, 0372, Norway; Institute for Molecular Medicine Finland (FIMM), HiLIFE, University of Helsinki, Helsinki, 00014, Finland; iCAN Digital Precision Cancer Medicine Flagship, University of Helsinki and Helsinki University Hospital, Helsinki, 00014, Finland; Drug Discovery and Chemical Biology (DDCB) Consortium, Biocenter, Helsinki, 00014, Finland; BioICAWtech, Helsinki, Helsinki, 00410, Finland

## Abstract

**Motivation:**

Drug–target interactions (DTIs) hold a pivotal role in drug repurposing and elucidation of drug mechanisms of action. While single-targeted drugs have demonstrated clinical success, they often exhibit limited efficacy against complex diseases, such as cancers, whose development and treatment is dependent on several biological processes. Therefore, a comprehensive understanding of primary, secondary and even inactive targets becomes essential in the quest for effective and safe treatments for cancer and other indications. The human proteome offers over a thousand druggable targets, yet most FDA-approved drugs bind to only a small fraction of these targets.

**Results:**

This study introduces an attention-based method (called as MMAtt-DTA) to predict drug–target bioactivities across human proteins within seven superfamilies. We meticulously examined nine different descriptor sets to identify optimal signature descriptors for predicting novel DTIs. Our testing results demonstrated Spearman correlations exceeding 0.72 (*P* < 0.001) for six out of seven superfamilies. The proposed method outperformed fourteen state-of-the-art machine learning, deep learning and graph-based methods and maintained relatively high performance for most target superfamilies when tested with independent bioactivity data sources. We computationally validated 185 676 drug–target pairs from ChEMBL-V33 that were not available during model training, achieving a reasonable performance with Spearman correlation >0.57 (*P* < 0.001) for most superfamilies. This underscores the robustness of the proposed method for predicting novel DTIs. Finally, we applied our method to predict missing bioactivities among 3492 approved molecules in ChEMBL-V33, offering a valuable tool for advancing drug mechanism discovery and repurposing existing drugs for new indications.

**Availability and implementation:**

https://github.com/AronSchulman/MMAtt-DTA.

## 1 Introduction

Developing novel pharmaceutical agents is a formidable endeavor, incurring expenditures amounting to billions of dollars and necessitating a prolonged time frame of 9–15 years for successful market introduction (Schuhmacher *et al.* 2023). Consequently, the pharmaceutical industry is increasingly motivated to explore alternative avenues, such as identifying new therapeutic applications for existing approved drugs. This strategic approach, commonly termed drug repurposing, holds considerable appeal due to its potential to accelerate drug development, curtail associated costs, and address unmet medical needs (Beijersbergen 2020). Drug–target interactions (DTI), i.e. the specific binding of drugs to target molecules that potently affect cellular functions are central to both drug discovery and repurposing efforts.

Six comprehensive, manually curated drug–target databases, namely, ChEMBL (Zdrazil *et al.* 2024), BindingDB (Gilson *et al.* 2016), DrugBank (Wishart *et al.* 2006), GtopDB (Harding *et al.* 2024), DrugTargetCommons (Tanoli *et al.* 2018), and DgiDB (Cotto *et al.* 2018) meticulously compile experimental bioactivity data spanning millions of compounds and thousands of protein targets. Despite their wealth of information, none of these repositories offers complete coverage of target interactions for approved drugs at the holistic proteome level. Therefore, several deep learning-based methods have been proposed to fill this gap (Abbasi *et al.* 2021).

Attention-based methods, as extension of deep learning, have gained major interest in drug discovery due to the breakthrough of the Transformers (Vaswani *et al.* 2017) in the field of natural language processing. In DTI predictions, attention mechanisms are used to learn numerical embeddings for compounds and targets, and to contextualize their complex relationships. MolTrans (Huang *et al.* 2021) is among the first methods to utilize parts of the Transformer architecture in DTI prediction, generating embeddings by applying an attention-based mechanism on one-hot encoded compound and target substructures. MolTrans operates primarily in the binary classification regime.

While MolTrans remains a strong benchmarking model, several approaches have addressed its limitations. Kang *et al.* (2022) introduced a regression method for predicting continuous binding affinity values. It generates embeddings via transfer learning from pre-trained Bidirectional Encoder Representation from Transformers (BERT) based methods (Devlin *et al.* 2018), where a compound’s Simplified Molecular Input Line Entry System (SMILES) string and a protein’s amino acid (AA) sequence are treated analogously to text in natural language. The use of pre-trained methods leverages vast amounts of unlabeled data. Subsequently, TransDTI (Kalakoti *et al.* 2022) explored a wider variety of pretrained methods for proteins, establishing embeddings generated by Evolutionary Scale Modeling (ESM) (Lin *et al.* 2023) and AlphaFold (Jumper *et al.* 2021) as effective alternatives for BERT-based embeddings.

Some methods extend the use of attention beyond generating separate embeddings for compounds and proteins. DTITR (Monteiro *et al.* 2022) and MCANet (Bian *et al.* 2023) successfully demonstrated how cross-attention can be applied across the descriptors of protein targets and compounds to gain additional context for the interactions. However, these methods rely solely on SMILES and AA sequences to generate embeddings, which, while practical, may oversimplify the complexities of structure and function. Some methods, such as Multi-TransDTI (Wang *et al.* 2022a), utilize additional descriptors alongside Transformer-generated embeddings, but still perform the prediction task with simplistic concatenations and fully connected layers without applying further attention.

Alternatively, MHTAN-DTI (Zhang *et al.* 2023) and DTI-GTN (Wang *et al.* 2022b) include interactions between drugs, proteins, diseases, and side effects in a graph format, using attention mechanisms tailored to graphs for promising DTI prediction results.

This work presents attention-based methods for DTI predictions in a regression setting for each of the seven superfamilies of the protein targets: enzymes, kinases, GPCRs, nuclear receptors, epigenetic receptors, ion channels, and transporters. These protein superfamilies are fundamental to cellular processes and disease mechanisms such as blood cancer and diabetes. (Evans and Mangelsdorf 2014, Lin *et al.* 2015, Moosavi and Ardekani 2016, Hauser *et al.* 2017, Cohen *et al.* 2021, Manolios *et al.* 2023). By targeting them, researchers can develop treatments that are highly specific, effective, and have fewer side effects.

Similar to the current state-of-the-art methods, our approach utilizes attention for generating compound and protein descriptors. However, instead of directly encoding SMILES and AA sequences, we use LASSO to select the best descriptors from various chemical fingerprints, physio-chemical properties of the compounds, and sequence-based and structural descriptors of the protein targets. We then employ sophisticated and flexible embedding methods for numerical descriptors (Gorishniy *et al.* 2022), and pass the embedded input through several layers of adjusted transformer encoder layers to learn the descriptors. We further focus on the combined compound-target features to understand their intricate relationships. As a result, we produce accurate DTI predictions using the powerful attention mechanism, while leveraging diverse and informative compound-target descriptor sets, paving the way for increasingly multimodal DTI prediction approaches.

In addition to evaluating our prediction methods using train–test split testing, we computationally validated our methods on a huge DTI dataset (185 676 bioactivities) from drug–target pairs provided by ChEMBL-V33 that were nonoverlapping with the training dataset. Finally, after building the prediction methods, we tested them to predict novel (previously untested) bioactive interactions for 3492 approved drugs in ChEMBL-V33, opening new avenues for drug repurposing. To our knowledge, no other comprehensive attention-based methods exist for the seven superfamilies of the druggable targets, trained and validated on such big datasets as proposed in this study.

## 2 Materials and methods

### 2.1 Training dataset

The training data in this project consists of bioactivity values between drug–target pairs. We utilize two continuous label types for bioactivity: DrugTargetProfiler (DTP) scores (Tanoli *et al.* 2020) and pChEMBL values from the ChEMBL database (Zdrazil *et al.* 2024). DTP scores range from 0 to 1, with 0 indicating inactivity. These scores are calculated based on bioactivity values, assay format and target family, thus mitigating the issues of heterogeneity often found in public databases like ChEMBL or BindingDB. The effectiveness of DTP scores was demonstrated by our success in the CTD DREAM challenge (Douglass Jr *et al.* 2022). The pChEMBL values, on the other hand, represent the negative logarithm of molar concentrations for various measures such as IC50, EC50, XC_50_, AC_50_, K_i_, K_d_, and potency. Higher pChEMBL values (e.g. >6) indicate more active interactions, while lower values (≤5) denote inactivity. We locally downloaded the two databases and extracted bioactivity labels by matching the drug–target pairs.

We collected the training data for active targets (pChEMBL >5, DTP >0) from DTP and ChEMBL, and verified inactive targets from PubChem (Wang *et al.* 2016) using their API. We considered those drug–target pairs as inactive for which pChEMBL ≤5, DTP = 0 and labeled as ‘Inactive’ at PubChem database.

Each data point includes both DTP scores and pChEMBL values as labels. The active dataset contained some duplicate drug–target pairs with differences in the recorded activity values, possibly due to different assay formats or detection methods. We handled this discrepancy by taking the median of the reported values both for DTP scores and pChEMBL values. After median filtering, we removed any pairs found in both the active and inactive datasets. Additionally, we removed inactive pairs where the DTP score was 0 but the pChEMBL value exceeded five. The resulting training dataset comprised 947 195 interactions between 452 296 compounds and 1251 targets. Within the compounds, 1884 are approved drugs, 120 in phase I, 268 in phase II, 336 in phase III, and the remainder are preclinical compounds.

We divided the full dataset into seven parts according to the respective target superfamilies. Finally, we sub-sampled 20% of each set for testing and kept the remaining 80% for training (i.e. one-time train–test split, instead of cross-validation). The label distributions of the training datasets are shown in Supplementary Figs S2–S4.

### 2.2 Workflow of proposed method

**Figure 1.**
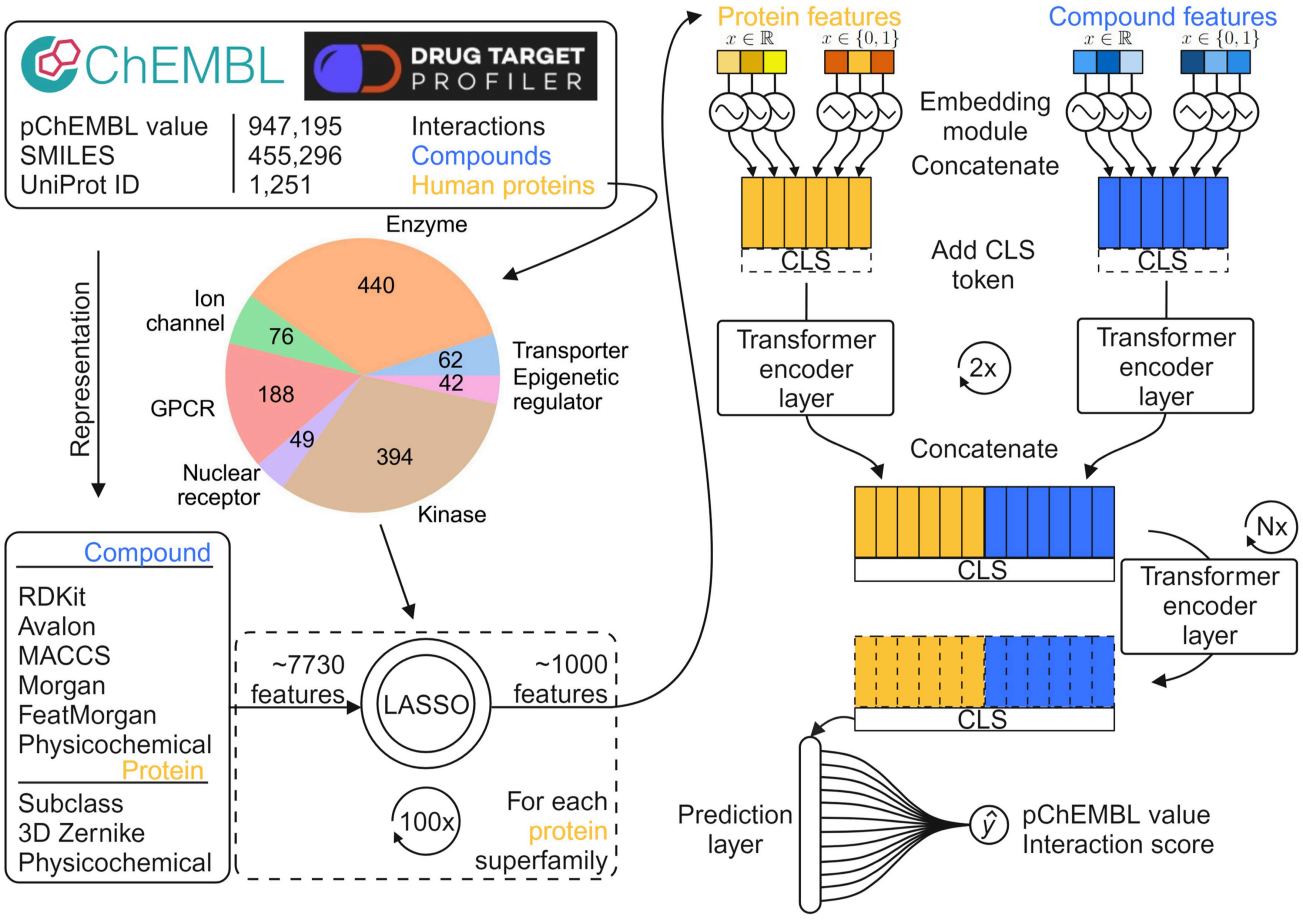
Workflow for the attention-based models to predict target bioactivities of approved drugs across seven protein superfamilies. Interaction labels are obtained from DTP and ChEMBL. We generated a high-dimensional descriptor vector from the data to represent compound–protein pairs. We selected the most relevant features with LASSO, resulting in one feature set for each protein superfamily. We repeat the selection 100 times for robustness and retain the most frequently chosen descriptors. We separate each set into protein and compound descriptors and pass them into specialized embedding modules. The embeddings are then concatenated, appended with a CLS token, and fed into a series of transformer encoder layers. Finally, the transformed CLS tokens are passed into a prediction layer to yield a pairwise binding affinity prediction. Each protein superfamily has two models, one to predict pChEMBL values and the other to predict DTP interaction scores (0–1).

Figure 1 presents the general workflow of our DTI prediction approach. After collecting and preprocessing the pairwise activities (pChEMBL values and DTP scores), we split the data into seven parts based on the respective protein superfamilies. We computed comprehensive, multimodal descriptor vectors for the compounds, which include chemical fingerprints and physicochemical properties. For the targets, we computed sequence-based descriptors, Zernike descriptors and subclass labels. To ensure the most informative descriptors are used, we apply LASSO-based feature selection separately for each of the seven protein superfamilies. The feature selection was based only on interaction score-based models for each superfamily separately. The compound–protein pairs and subsequently their embedding representations are the same in the DTP score and pChEBML value models, only their bioactivity labels are different. Thus, the selected descriptors are applicable to both types of models, and the two bioactivity labels are used to test the robustness of the models.

With the prepared datasets, we trained 14 attention-based models: one for predicting pChEMBL values and another for interaction scores, separately for each of the seven superfamilies. The architecture is uniform across all models, consisting of separate embedding modules for binary and continuous descriptors of both compounds and target proteins. These are followed by a series of transformer encoder layers. The encoder layers apply self-attention to the descriptor matrices, which are equipped with CLS tokens to learn the compound-target descriptors and their relationships. Finally, the CLS tokens are extracted and fed into a prediction layer that yields the final point prediction. The detailed model architecture is shown in Supplementary Fig. S1.

### 2.3 Attention-based architecture

We used an advanced deep learning architecture for our prediction problem. The right side of Fig. 1 shows the overall architecture, while Supplementary Fig. S1 illustrates the detailed model design. Our proposed model is a modified version of Feature Tokenizer Transformer (FT-Transformer) (Gorishniy *et al.* 2021), which aims to enhance the performance of neural networks when dealing with tabular data. It leverages the encoder part of the transformer model (Vaswani *et al.* 2017), relying heavil*y* on self-attention. Unlike most other attention-based models, the FT-Transformer can use numerical data in a tabular format as input, rather than text or image data. This allows our model to use descriptors beyond SMILES and AA sequences, while still benefiting from the self-attention process. Moreover, our model is flexible and can incorporate any numerical descriptors, facilitating future improvements as better compound or protein descriptors are discovered.

After obtaining the descriptor vector with selected features as an output from LASSO, we grouped them into four subvectors: Continuous compound descriptors (physicochemical descriptors), binary compound descriptors (fingerprints), continuous protein descriptors (sequence-based and Zernike descriptors), and binary protein descriptors (subclass labels), describing each compound–protein pair. The dimensions of the subvectors depend on the output from LASSO and vary between protein superfamilies. These four subvectors serve as inputs to our attention-based deep learning model. We separated continuous descriptors from binary descriptors to enable the use of specialized embedding schemes. Although embeddings are typically used for nonnumerical descriptors such as letters and words, we followed the unconventional approach of Gorishniy *et al.* (2022) and applied embeddings to the numerical descriptors. We used a periodic activation function combined with a fully connected layer and an activation function for continuous descriptors. The embedding module for a continuous descriptor x is as given by the following equation:


$${\mathrm{Embed}}_{c}(x)=\mathrm{ReLU}(\mathrm{Linear}(\mathrm{Periodic}(x)))$$

Here, ReLU and Linear refer to the Rectified Linear Unit and a fully connected layer, respectively. The periodic activation function (Periodic) converts a scalar input to a vector of arbitrary dimension, defined as:


$$\mathrm{Periodic}(x)=\mathrm{concat}[\text{sin}(v),\;\text{cos}(v)],v=[2\pi c_{1}x,\ldots ,2\pi c_{k}x],$$

where *x* is the scalar input, *k* is an arbitrary activation dimension and *ci* is a trainable parameter sampled from normal distribution *N*(0,σ), where σ is a hyperparameter to be tuned. This converts an input data vector $x\;\in R^{n}$ via periodic activation into a matrix $A\;\in R^{n\times 2k}$, making it suitable for the attention mechanism. For binary descriptors, the embedding module is a simple lookup table with a cardinality of two. Both embedding modules convert an *n*D vector into a tensor of size *n* × *d*, where d is a tunable embedding dimension. Importantly, the modules are independent descriptor-wise, meaning no weights are shared between embeddings of distinct descriptors.

After the embedding procedure, the model concatenates embedding matrices obtained from the continuous and binary descriptors while keeping the compounds and proteins separate. A CLS token row of initially random values is also appended to the embedding matrix. Originally introduced in the BERT architecture (Devlin *et al.* 2018), the CLS token facilitates learning comprehensive representations. The model then passes the resulting tensors through a series of consecutive Transformer encoder layers. We do not use positional encoding in the encoders, as the order of individual elements in descriptor sets does not matter. Self-attention is applied to compound and protein descriptors separately in the first two layers, after which they are concatenated into one complete matrix for the subsequent encoder layers to process together. The attention-based encoder layers form the core of our model architecture and greatly enhance its learning capabilities (Supplementary Table S8).

For the encoder layer structure, we followed the pre-norm variant of the FT-Transformer encoder, where the residual connection branches out before layer normalization. This differs from the original Transformer, where residual branching occurs after normalization, and it facilitates the optimization process during model training (Gorishniy *et al.* 2021). Additionally, we applied dropout to multi-head self-attention and fully connected layers in the encoder.

Following the encoder layers, we extracted the CLS tokens and discarded all the other rows in the data matrix. The encoder layers modify the CLS tokens via the attention mechanism so that they encapsulate sufficient knowledge and context about the compound-target descriptors. Thus, the CLS tokens contain enough information in a condensed form for the prediction module to use in the final interaction strength point prediction:


$$\hat{y}=\mathrm{Linear}(\mathrm{ReLU}(\mathrm{LayerNorm}(T_{\mathrm{CLS}}))),$$

where $T_{\mathrm{CLS}}$ refers to the CLS token. We conducted the training on 4 Nvidia Volta V100 GPUs and 40 Intel Xeon Gold 6230 (2.1 GHz) CPUs, parallelizing the training with the PyTorch Distributed Data Parallel framework and the Ray library functionalities. The training times for each model are recorded in Supplementary Table S1. We trained 14 predictive models, with each of the seven protein superfamilies having two separate models for pChEMBL values and DTP scores. We initialized the weights of linear layers with Kaiming initialization. For loss calculation, we used the logarithmic hyperbolic cosine loss function. The function is known to be less sensitive to outliers and is defined as given the following equation:


$$L\left(y,\hat{y}\right)=\log\left(\cosh\left(\hat{y}-y\right)\right),$$

where *y* is the true label, and $\hat{y}$ is the prediction. During preliminary testing, we observed that the log-cosh loss yielded better results than the commonly used mean squared error. For optimization, we opted for the AdamW optimizer.

We limited training to a maximum of 500 epochs and used early stopping with a patience of 10 epochs to avoid overfitting and preserve model generalizability. We checkpointed the model weights after each epoch, retaining five checkpoints with the lowest reported test losses, which we used as an ensemble for later predictions and inference.

### 2.4 Descriptor sets

Instead of relying on text-based descriptors generated by attention-based models, we analyzed various comprehensive sets of numerical descriptors reported in drug–target-based prediction methods in the literature. Some descriptor sets are related to compounds, whereas others are associated with target proteins. The following sections describe different descriptor sets adapted in the proposed method.

#### 2.4.1 Compound descriptors

All compound descriptors in the proposed study take as input the canonical SMILES representation for a compound. SMILES is a widely used system that notates the chemical structure of a compound in linear string format. We used the SMILES to calculate several fingerprints and physicochemical descriptors for the compounds in our training and test datasets.

##### 2.4.1.1 Chemical fingerprints

Chemical fingerprinting captures the identity of a molecule in a fixed-length, binary format based on various properties, such as shape and connectivity. Each bit in a binary fingerprint represents the presence (1) or absence (0) of a specific atom. The length of the bit vector varies depending on the fingerprint used. Fingerprints are used to compare or analyze the structural similarities between the compounds in methods known as quantitative structure-activity relationship (QSAR). Structurally similar compounds may exhibit similar activity towards a target (Cereto-Massagué *et al.* 2015). The Tanimoto or Jaccard coefficient ($T_{c}$**)** is a standard metric to compute structural similarity between fingerprints of two compounds. It is defined as $T_{c}=c/(a+b-c)$, where a and b are the number of atoms present in each compound fingerprint, and c is the intersection of atoms between the two compounds. The $T_{c}$ values range from 0 to 1, with higher values indicating greater structural similarity between two compounds.

Different fingerprint types may produce different similarity scores. This may result in so-called activity cliffs, where similar compounds exhibit different activity towards a target, thus going against the QSAR above assumption. To overcome this, studies have found it beneficial to incorporate several fingerprints for a more robust view instead of relying on only one scheme (Gorishniy *et al.* 2021, Periwal *et al.* 2022). We, therefore, adapted five different fingerprints in our work: Molecular Access System (MACCS) keys (Durant *et al.* 2002), RDKit (Landrum 2006), Avalon (Gedeck *et al.* 2006), Morgan and Featmorgan fingerprints (Rogers and Hahn 2010). MACCS keys are 166-bit-long vectors, where each bit corresponds to the presence or absence of pre-specified structural fragments. RDKit fingerprint is a path-based algorithm where molecule fragments are identified by following a molecular bond path and mapped to a fixed-size bit vector with a hash function. Avalon fingerprint uses a similar mechanism but considers slightly different molecule descriptors in encoding. Morgan fingerprint similarly uses a hash function but identifies the fragments by evaluating their atomic environment within a circular radius instead of a linear path. Featmorgan is a variation of Morgan that encodes different chemical properties of surrounding atoms. We used a radius of two for the Morgan and Featmorgan fingerprints, making them analogous to ECFP4 and FCFP4, respectively. The RDKit, Morgan and Featmorgan fingerprints used in this work are each 2048 bits long, while the Avalon fingerprint is 512 bits.

##### 2.4.1.2 Physicochemical descriptors for compounds

We used physicochemical properties to complement the molecular fingerprints in compound descriptors. Physicochemical properties refer to the physical and chemical characteristics that determine a compound's behavior in different environments and influence its interaction with target proteins. Using RDKit, we calculated a vector of 200 continuous, real-valued descriptors, each representing a specific physicochemical property. Examples of physiochemical descriptors include size, surface properties, partial charge, molecule fragmentation and drug-likeness.

#### 2.4.2 Descriptors for target proteins

Computing numerical descriptors for protein targets is a complex task with various existing methodologies (Yue *et al.* 2023). Descriptors based on protein 3D structure have become an attractive approach due to the increasing availability of 3D protein structures, complemented by the accurate predictions of AlphaFold (Jumper *et al.* 2021). A recent protein shape retrieval challenge (Langenfeld *et al.* 2020) highlighted the performance of several such descriptors, with a method based on 3D Zernike moments (Novotni and Klein 2004) consistently ranking among the top performers. We adopted this approach to represent the 3D structure-based descriptors for the protein surface as a numerical vector.

We obtained the protein 3D structures as Protein Data Bank (PDB) files to compute the Zernike descriptors. We collected experimentally validated structures for 1141 proteins using UniProt ID search and used AlphaFold high-confidence (>90) predictions for the remaining 270 proteins in this study. While using the binding pocket structure is known to yield more accurate results for certain protein classes, such as kinases (Cichonska *et al.* 2017), we used the whole protein structure for uniformity across the protein superfamilies. In addition to the Zernike descriptors, we used sequence-based descriptors to exploit patterns between individual proteins and further classified the proteins into subclasses.

##### 2.4.2.1 Zernike descriptors

Zernike descriptors are a series expansion of a protein surface as a 3D function. The surface function is expressed in spherical coordinates *f*(*r*, *θ*, *ϕ*) where ‘*r*’ is the radius, ‘*θ*’ is the polar angle, and ‘*ϕ*’ is the azimuthal angle. The protein is voxelized to obtain the functional form of the surface, and each atom is approximated by a Gaussian and integrated to obtain voxel points. The surface function is then expressed a*s*


$$f(r,\theta ,\phi )=\sum_{n=0}^{\infty }\sum_{l=0}^{n}\sum_{m=-l}^{l}C_{\mathrm{nlm}}Z_{nl}^{m}\left(r,\theta ,\;\phi \right)$$

where $C_{\mathrm{nlm}}$ are Zernike moment coefficients and${\;Z}_{nl}^{m}$ are 3D Zernike polynomials with order ‘*n*’, degree ‘*l*’ and repetition ‘m’. The polynomials are basis functions, defined as


$$Z_{nl}^{m}(r,\theta ,\phi )=R_{nl}(r)Y_{l}^{m}(\theta ,\phi )$$

where $Y_{l}^{m}(\theta ,\phi )$ are spherical harmonics and $R_{nl}(r)$ is a radial function:


$$R_{nl}(r)=\sum_{k=0}^{\frac{1}{2}(n-l)}N_{\mathrm{nlk}}r^{n-2k}$$

where *N* is a normalization factor. We find that the spherical harmonics *Y* depend on the angles *θ* and *ϕ*, while the radial function *R* only depends on the radius. By transforming the function ‘*f*’ to the Cartesian system: *f*(*r*, *θ*, *ϕ*) → *f*(*x*), the series expansion of the expression is given by


$$C_{\mathrm{nlm}}\;=\;\int_{|x|\leq 1}f(x)\bar{Z_{nl}^{m}}(x)dx,$$

Finally, the Zernike descriptors are obtained by taking the norm:


$$D_{nl}=\sqrt{\sum_{m=-l}^{l}{\left(C_{\mathrm{nlm}}\right)}^{2}}$$

These descriptors are rotationally invariant, meaning there is no need for pre-alignment of proteins before calculating the descriptors. The resolution of the surface representation, or the dimension of the descriptor vector, can be adjusted by altering the value of the order n. Based on literature (Kihara *et al.* 2011), we set n to 20, resulting in a 121D numerical vector. The Python code for calculating the descriptors, provided by Di Rienzo *et al.* (2022), was used in this study.

##### 2.4.2.2 AA sequence-based descriptors

To complement the 3D-based Zernike descriptors, we included sequence-based descriptors for proteins, resulting in a 576D feature vector. These descriptors are related to mono-peptide and bi-peptide amino acid frequency compositions, polarizability, hydrophobicity, and aromaticity. Scripts for computing these descriptors are available in our GitHub repository.

##### 2.4.2.3 Protein subfamily information

We used binary labels to further classify proteins into subfamilies with each superfamily, such as Tyrosine kinases (a subfamily of Kinase), Chemokine and Amine sub families (branches of GPCRs). This protein subfamily information is extracted from the ChEMBL database.

### 2.5 Feature selection using LASSO

Combining descriptors from the nine descriptor sets resulted in a total of 7730 descriptors, which would be computationally intensive to train on a dataset with approximately 1 million interactions. To address this, we performed feature selection to reduce the dimensions of the compound-target descriptor sets. We selected descriptors separately for each target superfamily using the same protocol. Effective feature selection retains the most important descriptors while discarding less significant ones, thus enhancing training efficiency and reducing memory requirements. It also improves the signal-to-noise ratio and mitigates overfitting (Saeys *et al.* 2007).

We used the Least Absolute Shrinkage and Selection Operator (LASSO) for feature selection. LASSO reduces the coefficients of less important variables to zero, eliminating the need to set arbitrary thresholds as required in methods like ridge regression. It also maintains interpretability by not altering the original form of the descriptors, unlike methods such as principal component analysis. Given our data format of (*x_i_, y_i_*), *i *=* *1, 2*,…, N*, the minimization objective of LASSO is defined as given by the following equation:


$$\mathrm{argmi}n_{\beta }\left\{\sum_{i=1}^{N}{\left(y_{i}-\sum_{j=1}^{M}\beta_{j}x_{ij}\right)}^{2}+\alpha \sum_{j=1}^{M}|\beta_{j}|\right\}$$

where *α* is a constant penalty term and (*β*_1_, …, *β*_*j*_) are the coefficients to estimate. We used a separate subset of data for each protein superfamily to solve the optimization problem. In the subsets, we included all DTP score-based interactions involving approved drugs and phase III investigational compounds with respective protein superfamilies within our dataset. Using subsets allowed for easier and faster convergence than using the full dataset. We used a concatenated vector of compound and target descriptors as the input representation for LASSO. As an exception, protein subfamily labels were excluded from the selection process and were thus always included in the final feature vector. The value for *α* was determined using an iterative fitting scheme along a regularization path with 5-fold cross-validation, performed separately for each protein superfamily. After finding the best *α*, we fit the model once again with the full subset and retained the features for which *β* > 0. Repeating the procedure on the same subset often resulted in a different *α* values and combinations of selected features due to data variance. For robust results, we conducted stability analysis by running the LASSO selection procedure 100 times, retaining the most frequently selected features. The subset of data was kept the same at each instance, only the data folding was randomized. This resulted in seven sets of features, one for each protein superfamily.

**Figure 2.**
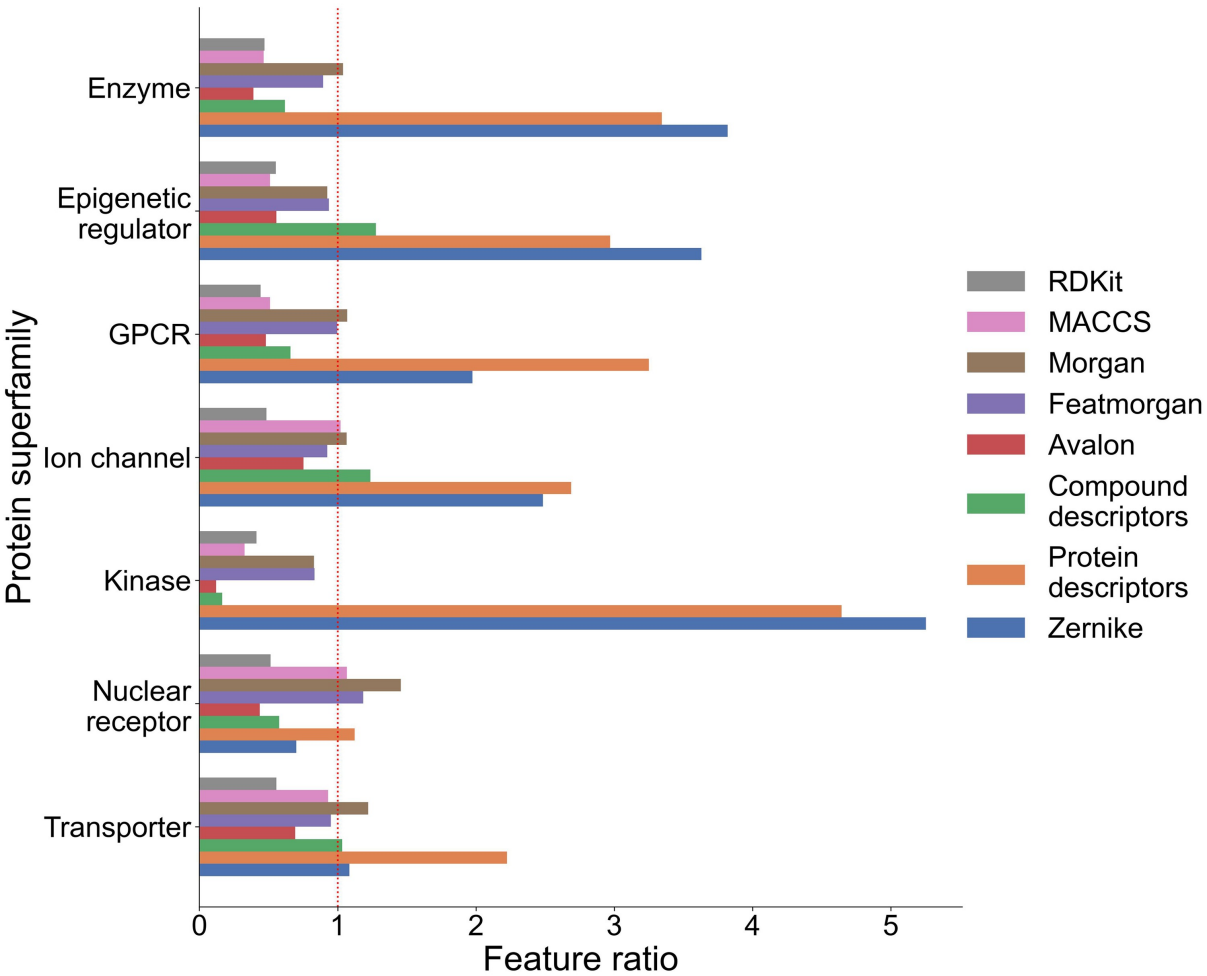
The change in descriptor vector dimension after feature selection. Originally, each compound–protein pair was represented by a 7719D vector (excluding protein subfamily labels). We reduced the dimension to 1000 with LASSO. Note that the selected descriptors are different across protein superfamilies. The horizontal bars show the direction of change in the dimension of separate descriptor sets to the 1000D descriptor vector for each superfamily when compared to the original, 7719D vector. Bars that cross the dotted reference line indicate an increased contribution of a descriptor set to the overall composition of a feature-selected vector than to the original vector. We omit protein subfamily labels from the comparison as they are always included in the descriptor vectors.

**Figure 3.**
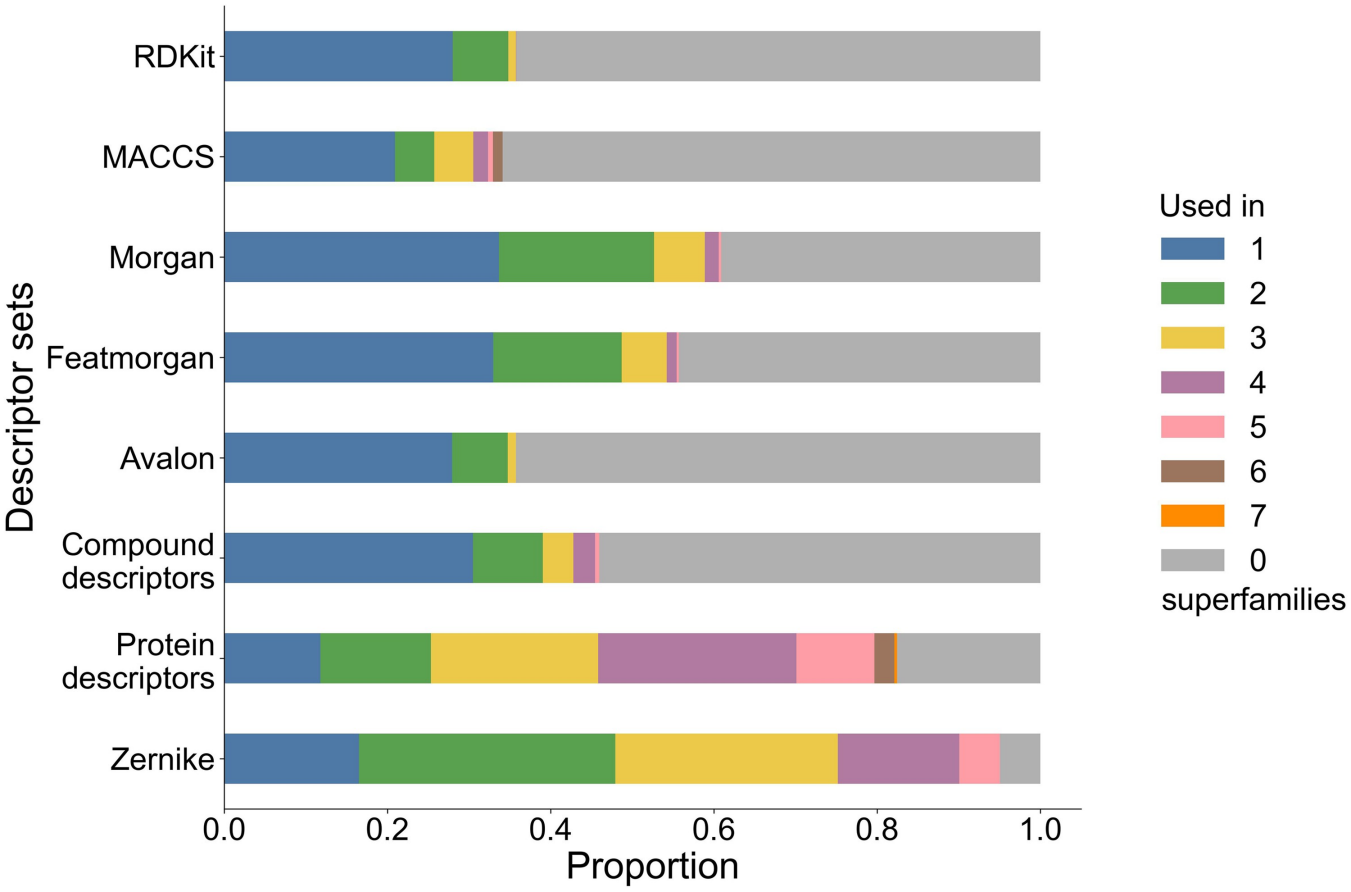
Proportions of how often individual descriptors belonging to a descriptor set are selected by LASSO into a model for each protein superfamily. The descriptors are grouped by their generality, i.e. how often they are selected for different superfamilies. The proportion of descriptors selected for all seven superfamilies is the lowest, while the proportion not contributing to any superfamily is the highest.

Figure 2 shows the superfamily-wise representation of selected descriptor sets. We fixed the number of descriptors at 1000 (excluding protein subclass labels), resulting in an approximately 8-fold decrease in descriptor counts. Using a descriptor vector of lower dimension resulted in performance degradation (Supplementary Table S7), while larger dimensions (>1500) led to GPU memory error during model training. Zernike and other protein-based descriptors were among the most important selected descriptors. Further analysis of descriptor set usage is shown in Fig. 3. Interestingly, nearly half of the compound descriptors are not used in any protein superfamily, and none of the compound descriptors were general enough to be used across all seven superfamilies. In contrast, protein descriptors were used more frequently.

### 2.6 Hyperparameter tuning

The hyperparameter optimization technique used in this work was Heteroscedastic Evolutionary Bayesian Optimization (HEBO) (Cowen-Rivers *et al.* 2022). We chose this method because it won the competitive NeurIPS black-box optimization challenge in 2020 (Turner *et al.* 2021). HEBO addresses two weaknesses found in plain Bayesian Optimization (BO) approaches. Firstly, BO often assumes neat, Gaussian noise likelihoods. Instead, HEBO takes into account data heteroscedasticity, meaning that it does not assume constant variance in the data. This is achieved with nonlinear input and output transformations. Secondly, BO usually utilizes one acquisition function that is assumed to be optimal. However, different acquisition functions can easily lead to conflicting results. Thus, HEBO uses an algorithm called multi-objective acquisition ensemble to search for the best solution via a Pareto front (Lyu *et al.* 2018). We combined HEBO with Hyperband (Li *et al.* 2018) to reduce computational costs by shutting down less promising runs early.

A simple train–test split was utilized in our hyperparameter optimization approach due to time and computational constraints. 80% of the data was used for training and 20% for testing. For each superfamily, we trained a model with initially random hyperparameters on the training split. The model was evaluated on the test split, and HEBO selected new hyperparameters, repeating the cycle until the budget limit was reached. The best hyperparameters were selected based on test set evaluation metrics, and the final model was tested on independent test data excluded from the optimization procedure.

Supplementary Table S2 lists the range of search spaces for each hyperparameter, as well as the constant hyperparameters. We set the budget for tuning at 144 h or 50 training iterations for each class, depending on which limit was reached first. We set the Hyperband grace period to 5 epochs, meaning that no iteration would be terminated before then. For a fair comparison, we kept the search spaces the same for all proteins, with the exception of the number of training epochs. We varied it between 50 and 75 depending on the observed speed of convergence and the size of the training set of a protein superfamily. Supplementary Table S3 shows the best resulting hyperparameter configurations for all the models.

### 2.7 Performance metrics

We evaluate our models with three metrics commonly used in DTI regression problems: root mean squared error (RMSE), Spearman's rank correlation coefficient, and concordance index (CI). Each metric gives a distinct insight to the model performance. Thus, it is important to consider them together as a whole, instead of focusing on only one stand-alone metric.

#### 2.7.1 Root mean squared error

RMSE measures the magnitude of error between predicted and actual activities, defined a*s*


$$\mathrm{RMSE}=\sqrt{\frac{\sum_{i=1}^{N}(y_{i}-\hat{y_{i}})}{N}}$$

where *y* is the true label, $\hat{y}$ is the prediction and *N* is the total number of datapoints tested. A lower RMSE value indicates a better fit of the model to the data.

#### 2.7.2 Spearman correlation

Correlation describes how well the relationship between the predicted and true labels can be described using a monotonic function, i.e. if the predicted values increase or decrease in tandem with the labels. It does not assume a linear relationship between the variables and is thus less sensitive to outliers. The correlation produces values between −1 and 1, where −1 indicates perfect inverse monotonic, 1 perfect monotonic, and 0 no monotonic relationship. Spearman correlation is defined by the following equation:


$$r_{s}=1-\frac{6\sum_{i=1}^{N}{\left\{R\left(y_{i}\right)-R(\hat{y_{l}})\right\}}^{2}}{N(N^{2}-1)}$$

where *R*(·) is the rank of a variable. In the context of our study, higher Spearman correlation is better.

#### 2.7.3 Concordance index

Concordance index (CI) refers to the likelihood that, when randomly selecting two drug–target pairs, the predictions made on those pairs are in the correct order in terms of label value. CI is defined using the following equation:


CI=1Z∑yi>yjhy^i-y^j, hu=1, u>00.5, u=00, u<0,

where *Z* is the number of distinct label values in the data. An ideal model achieves a CI of 1.0, while predictions obtained by random guesses will yield a CI of 0.5.

## 3 Results and discussions

### 3.1 Model training and testing

We assessed the performance of the proposed methods with a testing dataset separated from the original data prior to training. The train–test split was obtained by randomly sampling 20% of the full data for testing and hyperparameter tuning, while the remaining 80% was used for training. The approach is more resource-efficient compared to other common techniques, such as *k*-fold cross-validation. The random sampling resulted in near-identical bioactivity distributions between training and testing sets for each superfamily (Supplementary Figs S2 and S3) because of the large size of the original, unsampled dataset. We conducted the data-splitting process separately for each protein superfamily.

**Figure 4.**
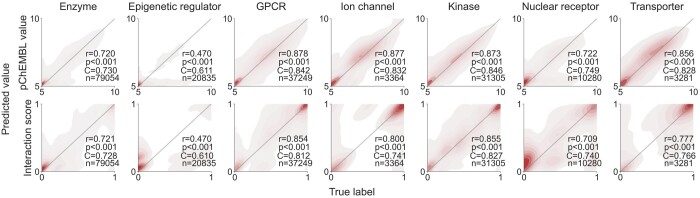
Contour plots of the testing results for each protein superfamily. The top and bottom rows correspond to models predicting pChEMBL values and interaction scores, respectively. The gray diagonals represent the ideal results, where predictions match perfectly with the true labels. Each plot is annotated with the Spearman correlation coefficient (*r*), the associated *P*-value (*P*), the concordance index (*C*), and the number of interactions (*n*).

Figure 4 shows the testing results and contour plots for predicting pChEMBL values and interaction scores. The results are based on a mean of predictions from five model checkpoints of different weights. The checkpoints are obtained by saving model weights after each epoch during a training run, and then selecting those with lowest reported test losses for use in inference and prediction. This ensemble approach slightly improved the performance without adding overhead to the training. Although the prediction times do increase with the ensemble, they remain sufficiently low. We also reported average prediction time for an interaction across each of the seven superfamilies in Table 1. The times vary mainly due to differences in the model sizes.

**Table 1.** Testing results from final training after hyperparameter tuning.^a^

Protein family	Spearman	RMSE	CI	Prediction time for one pair (ms)
Enzyme	0.720	0.509	0.866	34.6
Epigenetic regulator	0.470	0.560	0.811	16.1
GPCR	0.878	0.679	0.865	34.2
Ion channel	0.877	0.644	0.875	32.9
Kinase	0.873	0.625	0.861	37.7
Nuclear receptor	0.722	0.779	0.822	9.1
Transporter	0.856	0.696	0.848	35.2

a The results are derived from an ensemble of five best checkpoints with the best test results in each training run. We report the time taken to predict one drug–target interaction with each ensemble in milliseconds.

The test results imply that the model design leads to strong learning capabilities across protein superfamilies. However, an exception is observed in the model for epigenetic regulators. As seen in the tail of the contour plot in Fig. 4, the epigenetic regulator model tends to underestimate the activity of compounds, often predicting them as inactive. A similar trend is visible in the nuclear receptor model and, to some extent, in the enzyme model. We attribute this behavior to the skewness of the training data label distribution towards inactive values (as shown in Supplementary Fig. S2). For the other models, the predictions align well with the true labels.

### 3.2 Model validation on independent test set

The testing results alone are insufficient to evaluate model performance. Testing reveals a model's general behavior and learning capacity, but the results can be overly optimistic. This is partly because the test set is also used for hyperparameter tuning, meaning that the model is optimized to make predictions on the training dataset. This issue is often solved by splitting the test set into two sections, one for tuning and the other for testing. However, even with this technique, the prediction setting remains too lenient for practical performance evaluation, as the compounds and proteins in the test set likely overlap or are similar to those used in the training data.

To obtain a more objective understanding of our models' robustness and usefulness, we collected a new, independent validation dataset. The validation set includes those compound-target pairs present in ChEMBL-V33 reported in articles/documents not considered in the train–test split sets (Supplementary Fig. S5). Similar to the training data, some drug–target pairs contained multiple conflicting entries (possibly due to different assays for detection technologies being used). We retained the median label value of such cases and discarded the rest. Furthermore, we removed interactions for some proteins for which there were no Zernike descriptors available. The resulting validation set consists of 185 676 interactions between 119 034 compounds and 1681 targets. This independent validation set is provided in Supplementary File S1 to help reproduce our comparative analysis.

**Figure 5.**
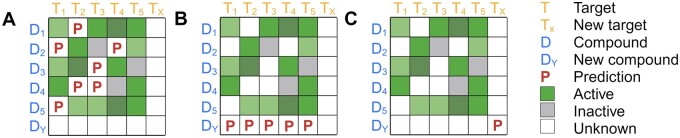
An illustration of the three validation scenarios. (A) bioactivity imputation, where all validated compounds and targets are available in the training set, (B) new compounds, where none of the compounds but all targets are available in the training set, (C) new compounds and new targets, where neither the compounds or proteins are available in the training set.

With this external validation set, we designed three validation scenarios of increasing difficulty, as shown in Fig. 5. The first and easiest scenario is bioactivity imputation, where the validation set contains compounds and proteins already present in the training set; only the compound-target pairings are different. The second scenario includes interactions between unseen compounds and seen proteins. Finally, the third and most challenging scenario contains only compounds and proteins not seen in the training set. We omitted the apparent fourth case of known compounds and unknown proteins due to inadequate data collection.

**Figure 6.**
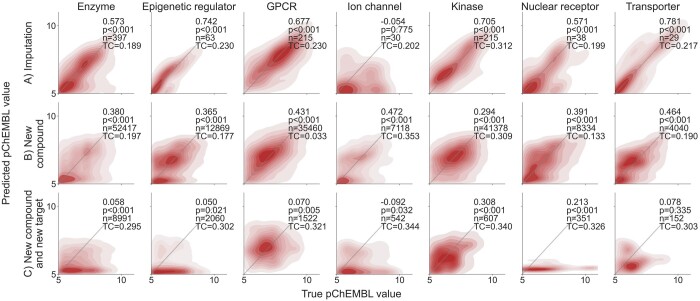
Contour plots of the validation results for each protein superfamily in three scenarios. The results are based on the pChEMBL labels. The gray diagonals represent the ideal scenario, where predictions match perfectly with the true labels. Each plot is annotated with the Spearman correlation coefficient and the associated *P*-value, the number of data points (*n*) and the average Tanimoto similarity (TC).

We evaluated the performance in the three independent validation scenarios across seven superfamilies, using an ensemble of the five best checkpoints, similar to the testing results. Figure 6 displays the contour plots, the Spearman correlations and their associated *P*-values, the number of pairwise interactions, and the average Tanimoto coefficients (TC) for each protein superfamily and validation scenario. Spearman correlation and RMSE are also reported in Supplementary Table S4.

We calculated the average TC by applying the similarity equation to find the similarity between all compounds in the training and validation sets and taking the mean of the resulting similarities. We used RDKit fingerprints for molecule representation, noting that different fingerprints would likely result in slightly different similarity scores.

Unexpectedly, we observed low TC in the bioactivity imputation validation scenario. This apparent discrepancy of shared compounds and low structural similarity can be explained by the relatively low number of compounds in the bioactivity imputation datasets. Consequently, the presence of the same compounds between the compared sets has a minimal influence on the overall similarity score of the sets. In general, compound similarities are low across validation sets (TC < 0.35), which increases the difficulty of predictions in the validation set.

As expected, the performance generally decreases as the test cases become more challenging. The models performed well in bioactivity imputation, with the exception of the ion channel model. Notably, the epigenetic regulator performed surprisingly well compared to the train–test results shown in Fig. 4. We note that the low number of available data points in the bioactivity imputation scenario leaves room for variations in results due to chance. This is in contrast to the new compound validation scenario, where the datasets are sufficiently large. Despite the decrease in model performance, the overall results in this second scenario are satisfactory. We confirm an adequate predictive power across protein superfamilies when identifying new compounds for known targets.

The third prediction scenario remains too challenging for most superfamily models. Interestingly, the only exception is the kinase model, where the performance surpasses that of the new compound’s validation scenario. We hypothesize that the inherent similarity between kinase structures diminishes the effect of unknown proteins, making the last two scenarios of similar difficulty for kinases. For the other models, predicting the interaction between an unknown compound and an unknown protein is more difficult. This is a common phenomenon in contemporary DTI prediction methods, as this validation scenario is practically difficult for current methodologies and is mostly ignored in existing studies (Tan *et al.* 2021).

### 3.3 Comparison with other methods

Davis (Davis *et al.* 2011) provides large-scale benchmarking data from high throughput target activity screening, where each of the 442 human kinases is screened against each of 72 inhibitors (442×72 = 31 824 interactions). While the Davis dataset is widely used in DTI research, its exact use in model evaluation differs between studies. For a fair comparison, we closely follow the testing pipeline devised by Monteiro *et al.*, the authors of DTITR (Monteiro *et al.* 2022). Released in 2022, the DTITR method is relatively similar to ours, with the most significant difference being their use of encoded AA sequences and SMILES as input instead of curated features.

Monteiro *et al.* filtered the Davis data by only including proteins with residue lengths between 264 and 1400 and compounds with SMILES string lengths between 38 and 72 characters, resulting in 423 protein targets and 69 compounds. They then split the data into six folds, five used for training and hyperparameter tuning. The sixth fold was used for testing and comparison against other models. We utilized the same data and train–test folds, except for the further exclusion of eight proteins for which Zernike descriptors could not be calculated and the removal of a few duplicates. We used the same 3D structure for proteins with point mutations as for the corresponding wild-type proteins. This is because we were unable to find structures for mutant proteins. Subsequently, we repeated the training workflow for the kinase model based on the provided training folds, including feature computation and selection, hyperparameter tuning, and training. With near-identical training and testing data between models, the results can be attributed solely to differences in modeling approaches.

**Figure 7.**
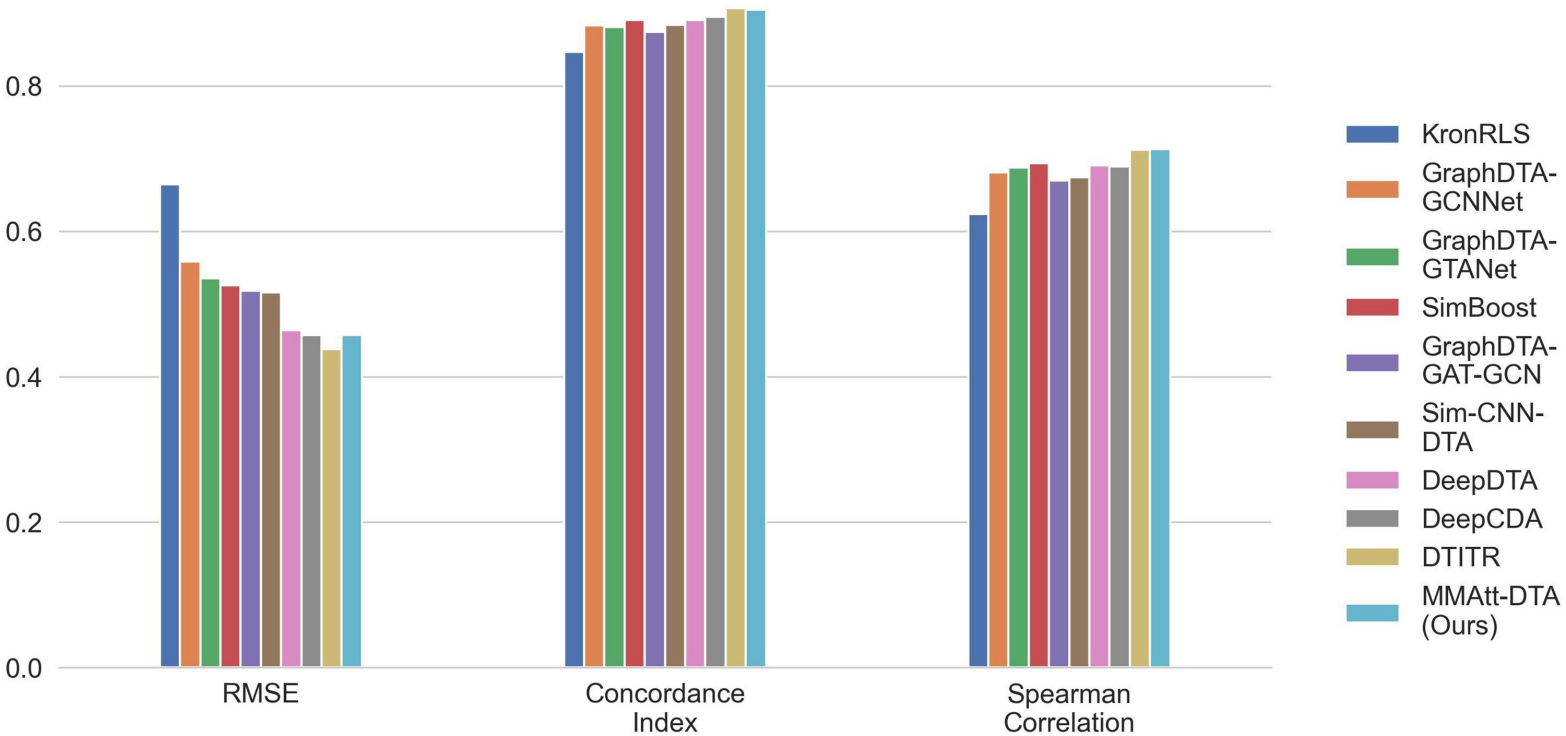
State-of-the-art comparison on the Davis dataset with three performance metrics. The results are derived from predictions on the test split constructed by authors of the DTITR method (more details in Supplementary Table S5).

Figure 7 displays the results obtained from model predictions on the test fold (see details in Supplementary Table S5). In addition to our model and DTITR, the tested models are KronRLS (Pahikkala *et al.* 2015), GraphDTA and some of its variants (Nguyen *et al.* 2021), SimBoost (He *et al.* 2017), Sim-CNN-DTA (Shim *et al.* 2021), DeepDTA (Öztürk *et al.* 2018), and DeepCDA (Abbasi *et al.* 2020). All these comparison models have been trained on the same data by Monteiro *et al.* for consistency. Likewise, the reported results arise from predictions on the same test fold. Our model performs similar to DTITR, while outperforming the other models in terms of Spearman correlation and concordance index. DTITR does maintain a slight lead in terms of RMSE, with our model achieving the second-best performance together with DeepCDA. Out of the many possibilities, one reason could be that our model is currently unable to clearly distinguish between mutated and wild-type proteins in the Davis kinase dataset, unlike the DTITR method. Additionally, adhering to the precise training scheme and data folds dictated by the DTITR authors may put us at a slight disadvantage. This is because DTITR’s model may be optimized for the data fold settings they established.

We provide further comparisons with results reported by more recent methods published in 2023–2024: TransVAE-DTA (Zhou *et al.* 2024), GraphCL-DTA (Yang *et al.* 2024), Multidta (Deng *et al.* 2024), GEFormerDTA (Liu *et al.* 2024), and GLCN-DTA (Qi *et al.* 2024) (Supplementary Table S6**)**. The results were obtained directly as reported in the articles. However, we ensured that the methods used a cross-validation scheme based only on the Davis dataset, like our comparison in Fig. 7. We observed that our model surpasses the other models both in terms of CI and RMSE. We excluded the Spearman correlation metric as it was not reported in most of the studies. Overall, we reached a level of performance comparable to the current state-of-the-art.

### 3.4 Predicting novel bioactive interactions for approved drugs to open new avenues for drug repurposing

**Figure 8.**
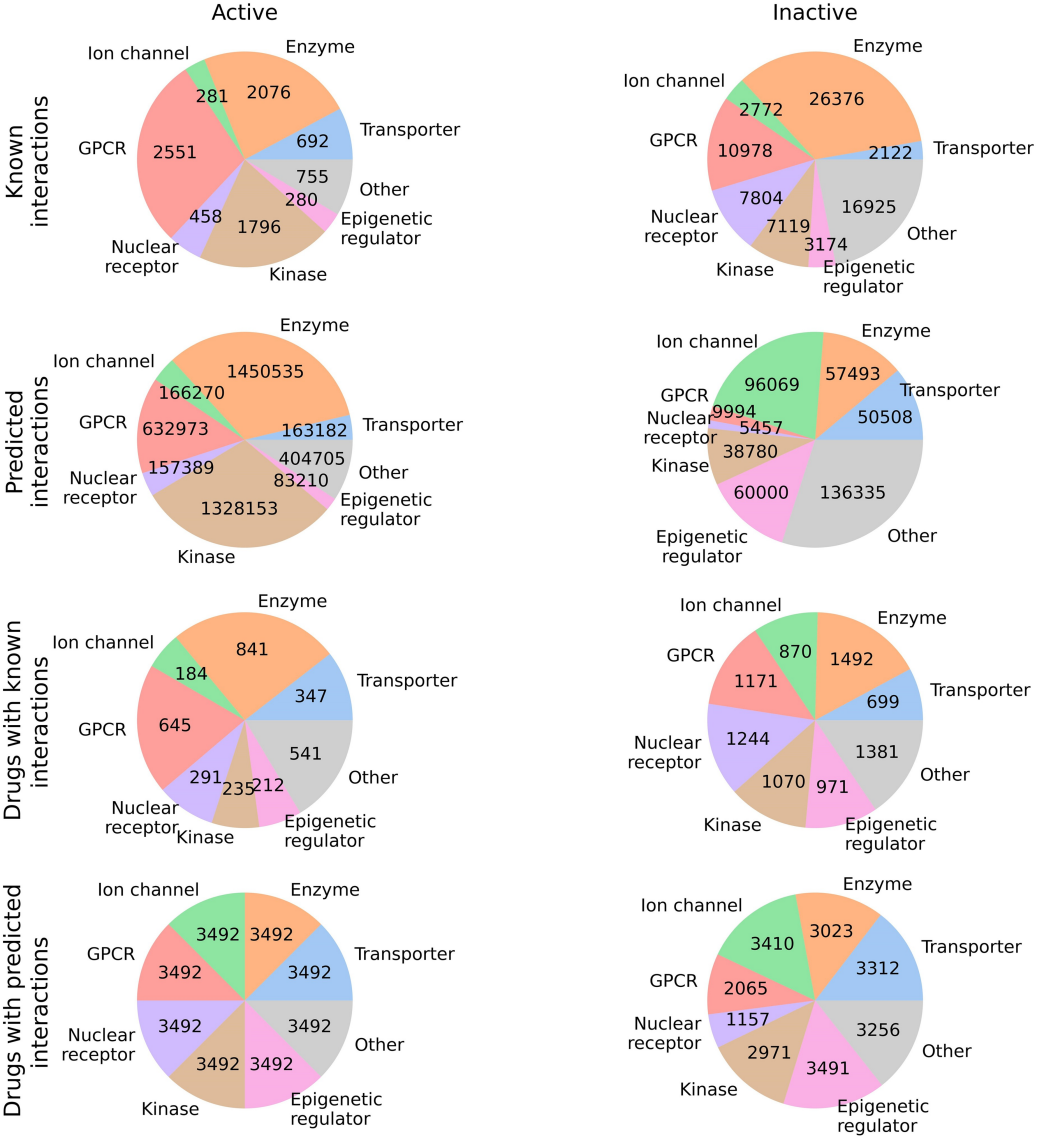
The number of known and predicted interactions and number of approved drugs for each protein class. The proportions are separated into active and inactive categories. An interaction with pChEMBL value of 5.0 or smaller is considered as inactive. For the predicted values, we set the inactivity threshold at 5.1 due to the regression model predicting many values close to, but not exactly, 5.0. Supplementary File S2 shows more details on the predicted and known interactions of approved drugs across 1411 human proteins.

After successfully evaluating the robustness of the proposed prediction models using testing and validation datasets, we used the deployed models for each of the seven protein superfamilies to predict new and previously untested bioactive interactions for 3492 approved drugs against 1411 human targets. Figure 8 shows the distribution of known and predicted interactions across seven protein superfamilies. More details on the predicted and known interactions in terms of pChEMBL values are available in Supplementary File S2. Prediction of secondary active and inactive targets for approved drugs can lead to new drug repurposing applications and may become beneficial for finding new treatments for various diseases.

## 4 Conclusions and future directions

In this study, we proposed attention-based methods to predict novel DTIs across seven superfamilies of protein targets. Attention-based methods are the latest extension of deep learning and are proven to perform better than conventional machine learning or deep learning methods. Our prediction models for six of the seven superfamilies achieved high Spearman correlations (>0.7), except for the epigenetic regulators. The low performance for targets from epigenetic regulators superfamily is likely due to the skewed training data distribution towards inactive values. We also compared our proposed method with fourteen machine learning, deep learning and graph-based methods reported in the latest literature for DTI prediction on the Davis kinase activity dataset, demonstrating state-of-the-art performance. We provided a benchmark training dataset of 185 676 interactions across 119 034 compounds and 1681 human targets. This comprehensive benchmark dataset (Supplementary File S1) can be utilized to develop and test future drug–target prediction methods.

Importantly, we also performed comprehensive computational validation on the non-overlapping drug-target pairs from ChEMBL-V33. This comprehensive dataset contains 185 676 interactions, 119 034 compounds and 1681 targets not available in the training dataset. We defined three scenarios to assess the validation results, as shown in Fig. 5. Except for ion channels, prediction models for most of the protein superfamilies performed well in two scenarios: missing value imputation (Spearman correlation > 0.57) and new unseen compounds (Spearman correlation > 0.36). For the third and most challenging scenario (new unseen compound and targets), kinase prediction models showed Spearman correlation > 0.3. This third scenario is not usually reported in DTI prediction studies. With such a high performance in testing and validation, the proposed prediction methods are expected to be generalizable to predict interactions with new compounds across seven protein superfamilies.

The proposed methods should be particularly helpful during the hit identification phase in target-based drug discovery. We provided all the training, testing and validation datasets, source codes, and user guides on GitHub (https://github.com/AronSchulman/MMAtt-DTA), so that researchers can easily reuse our methods or extend our analysis. Additionally, we utilized the deployed methods to predict novel and previously unexplored interactions for 1411 human proteins across the seven superfamilies with 3492 approved drugs (with max phase of 4 in ChEMBL-V33). We completed the DTI matrix of size 3492 × 1411 with the help of known and predicted pChEMBL values (Fig. 8, Supplementary File S2). This complete DTI matrix for approved drugs can open new avenues of drug repurposing and help understand their mechanism of action. Furthermore, the predicted bioactivities can enhance the accuracy of in-silico drug combination prediction algorithms by incorporating predicted DTIs as feature vectors when training the prediction models.

In the future, we plan to explore language-based embeddings using SMILES and AA strings to investigate whether these can further improve our approach. Some larger protein superfamilies can be divided into smaller groups; e.g. enzymes could be further divided into transferases and cytochromes. Similarly, new protein superfamilies could be added, such as surface antigens, secreted and structural proteins and membrane receptors (other than GPCRs). The robust performance demonstrated across the protein superfamilies suggest that our attention-based approach can be extended to other superfamilies and subfamilies, provided sufficiently large training datasets are available for target activities.

## Supplementary Material

btae496_Supplementary_Data
