# Supplementary material for: Attention-based approach to predict drug–target interactions across seven target superfamilies
Source: Bioinformatics. 2024 Aug 8;40(8):btae496. doi: 10.1093/bioinformatics/btae496 (PMC11520408; doi:10.1093/bioinformatics/btae496)

***
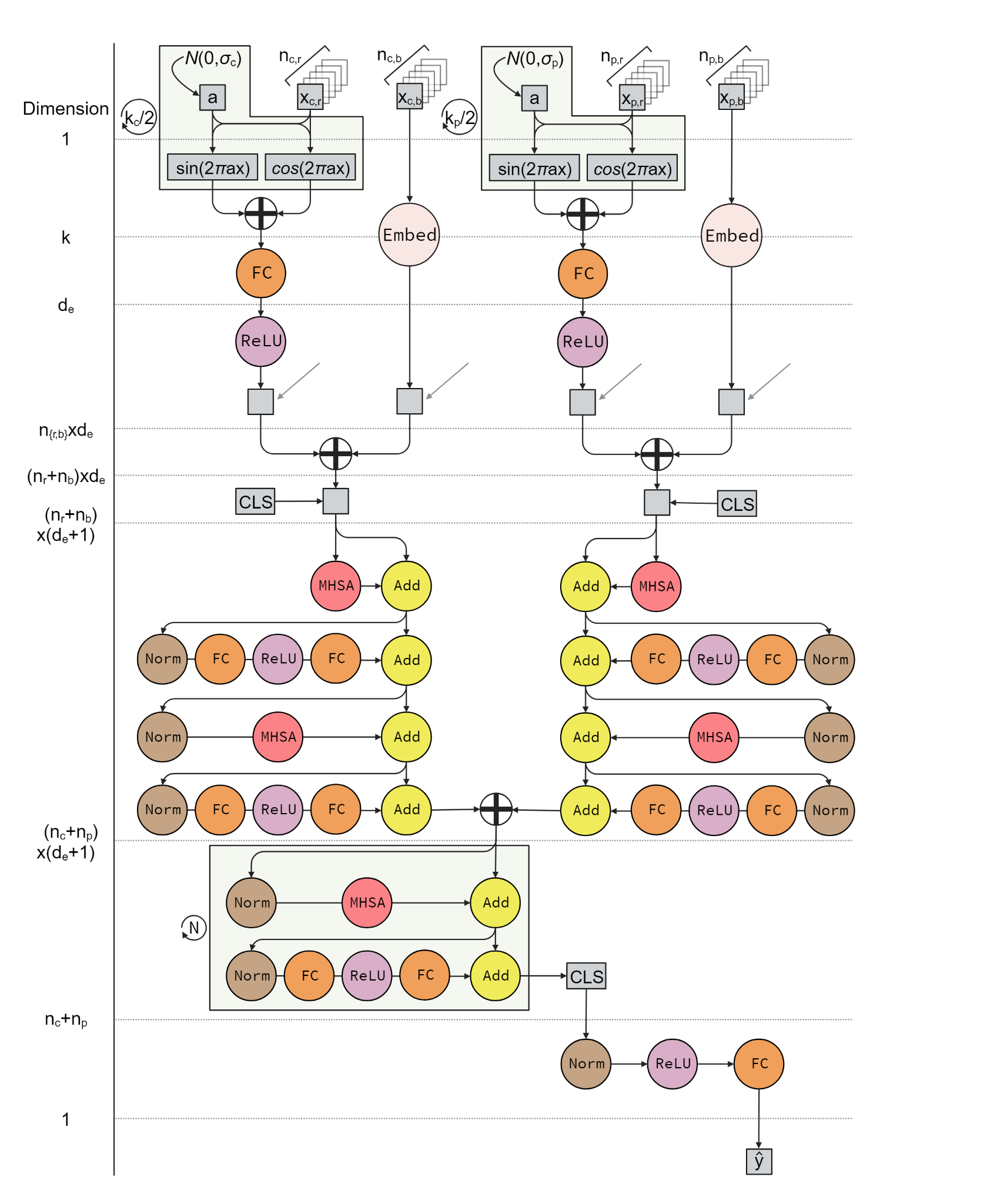
***

**Supplementary Figure 1:** Detailed architecture of the model. The ‘+’ sign indicates tensor concatenation. The column on the left of the figure shows tensor dimensions, where $k$ is periodic activation dimension, $d_{e}$ is embedding dimension, and $n_{c}$, $n_{p}$, $n_{r}$ and $n_{b}$ are the number of compound, protein, continuous and binary features, respectively. A given dimension applies each time a dashed line intersects with an arrow.


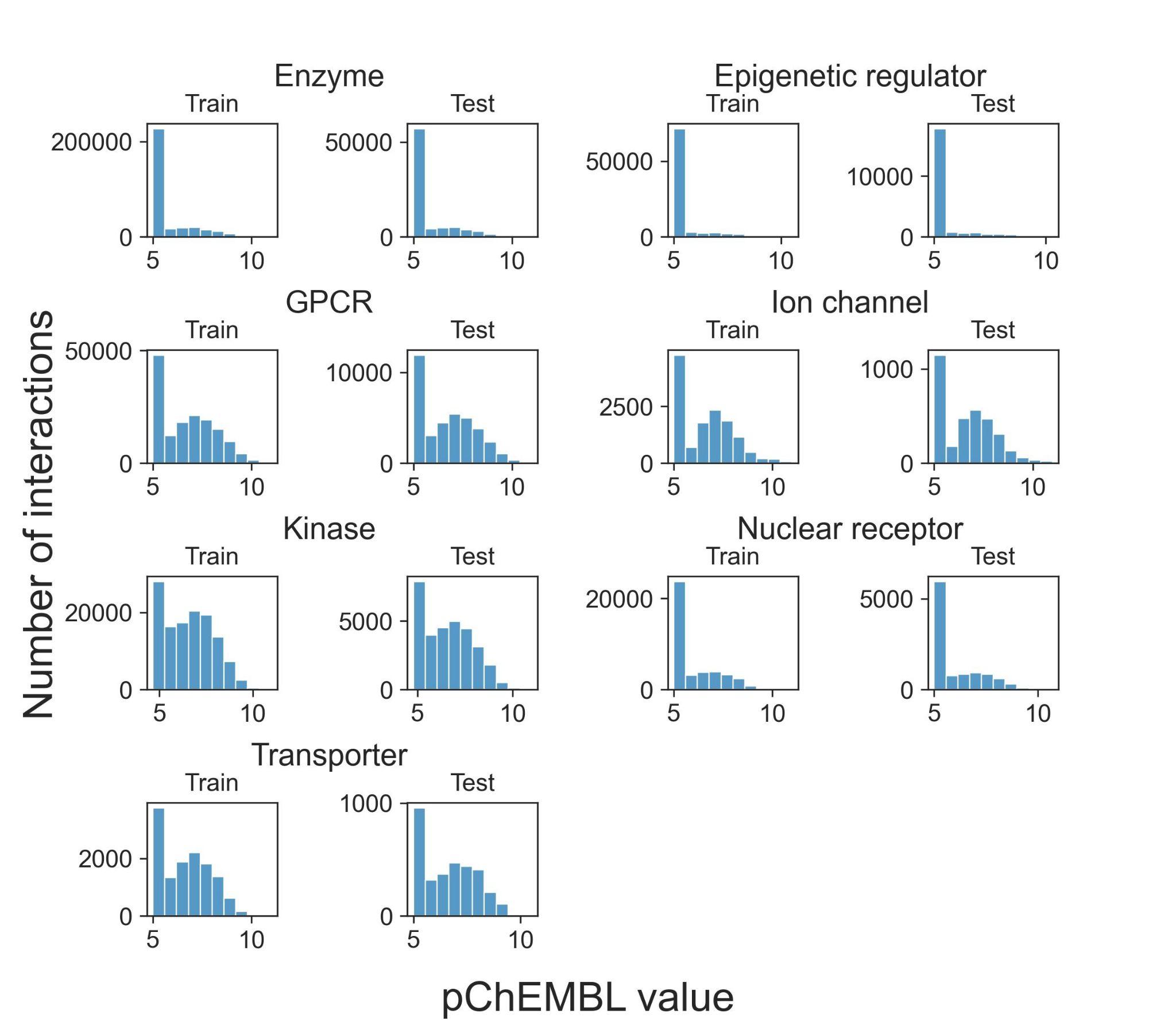


**Supplementary Figure 2:** Bioactivity distributions in the training and test data for each protein superfamily. The Y-axis represents the total number of interactions and the X-axis represents pChEMBL values of interactions (range: 5-11). Five indicates non-potent interactions (inactive). The pChEMBL values were extracted from ChEMBL database.


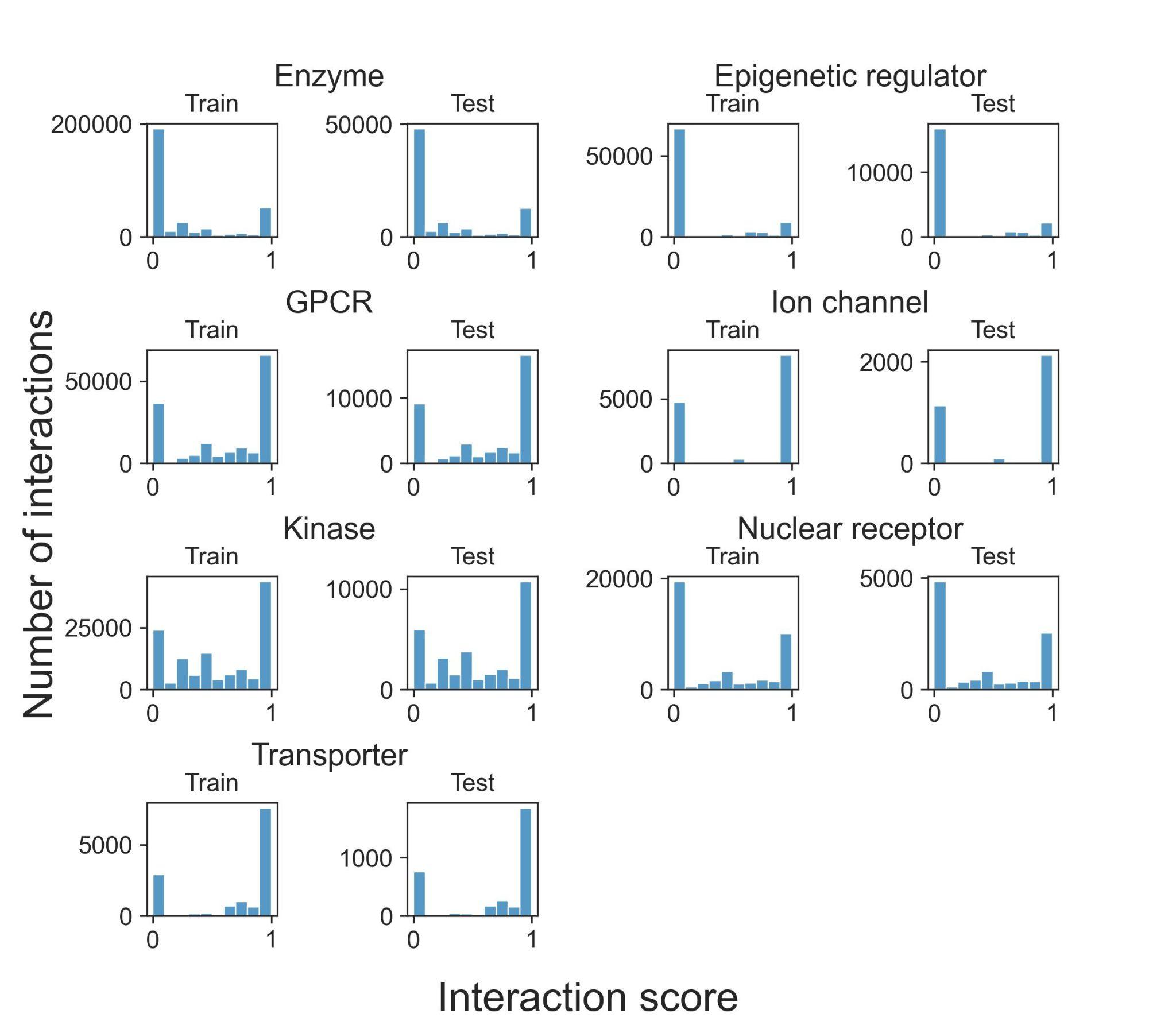


**Supplementary Figure 3:** Bioactivity distributions in the training and test data for each protein superfamily. The Y-axis represents the total number of interactions and the X-axis represents interaction scores from DTP (range: 0-1). Zero indicates non-potent interactions (inactive). The Interaction scores were extracted from DTP database.


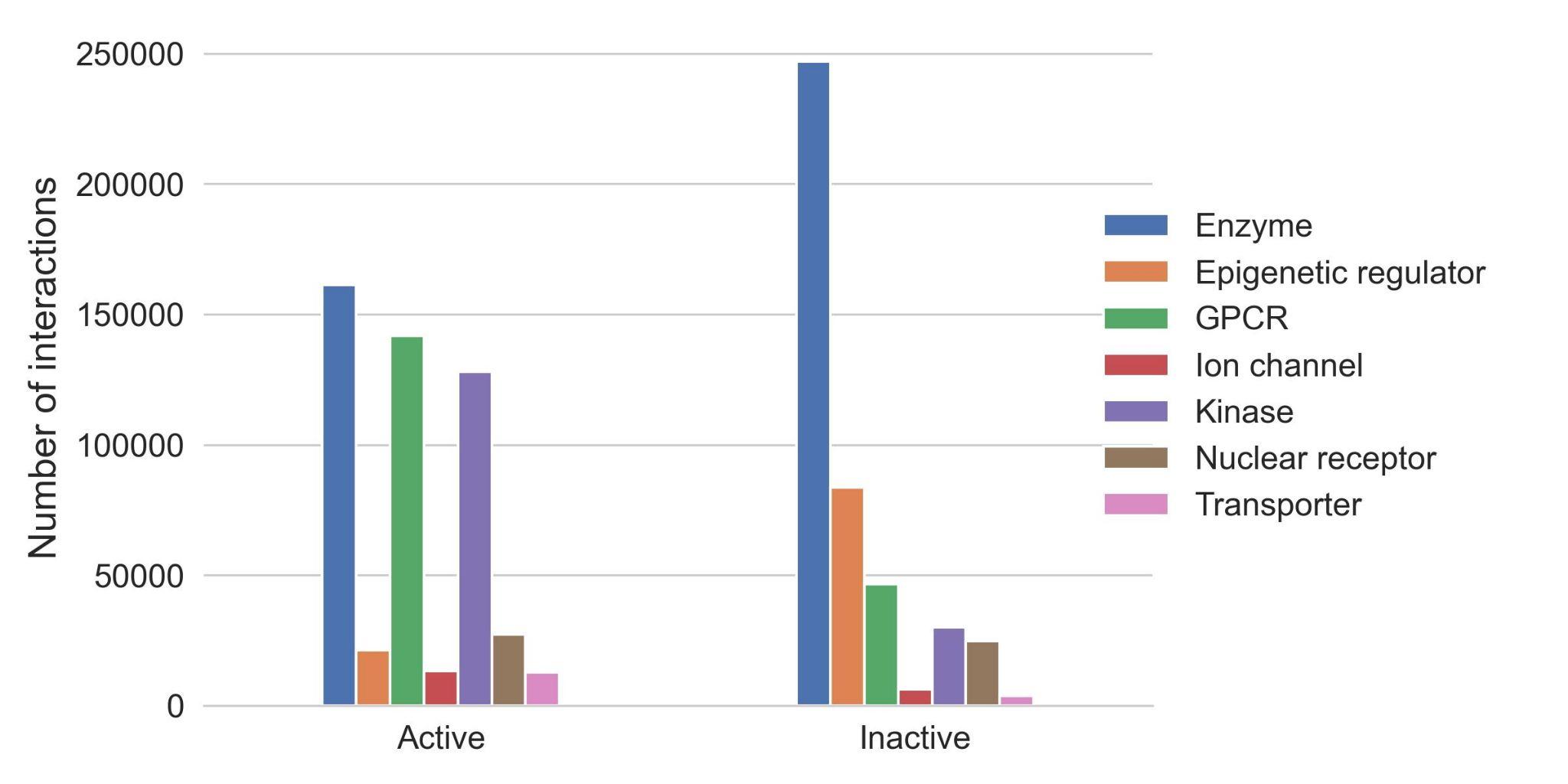


**Supplementary Figure 4:** The distribution of the binarized bioactivity values in the unsampled training data for the seven protein superfamilies. Here, a pChEMBL value of 5 or less is considered inactive and a pChEMBL value of more than five as active. For the model training and testing, we use continuous bioactivity values.

.
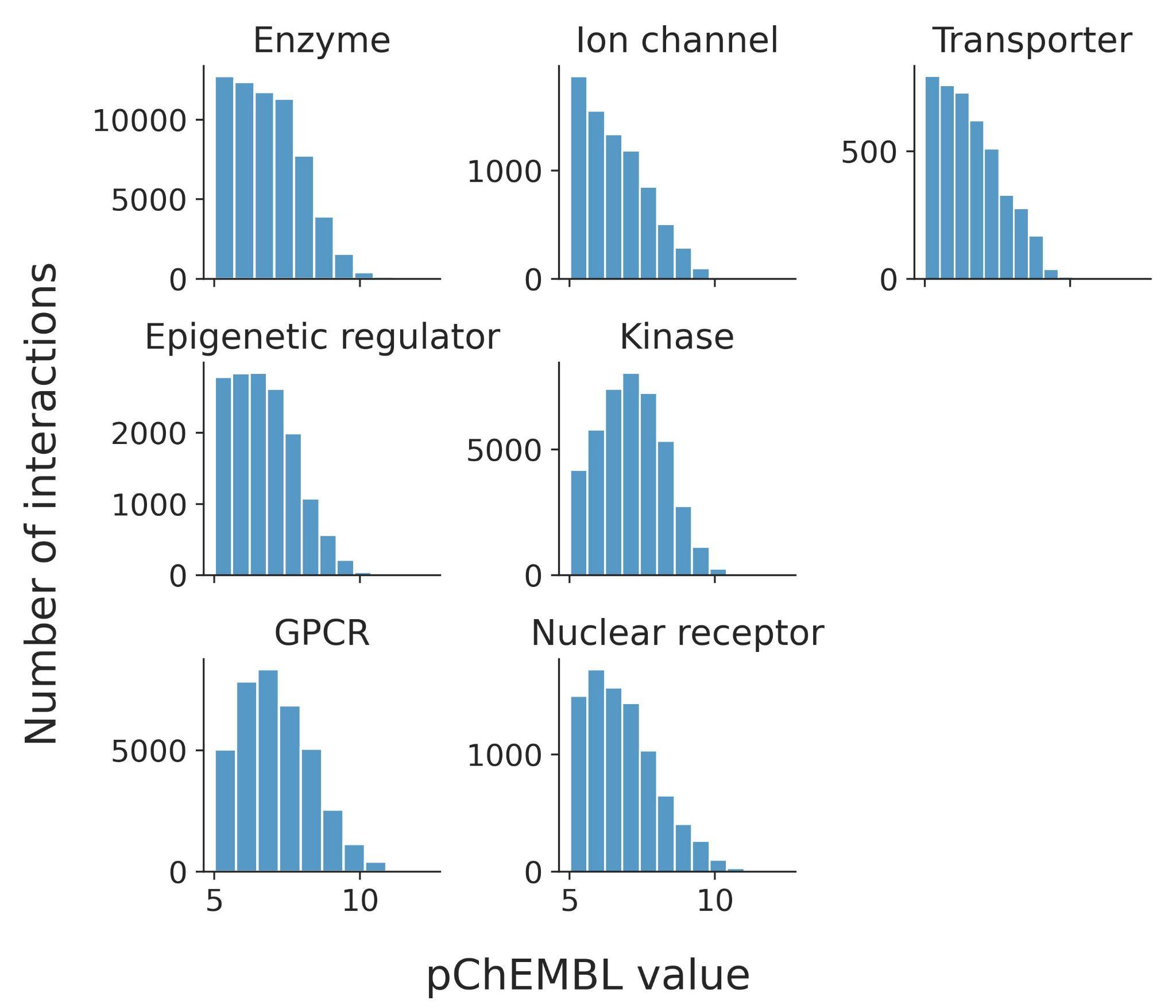


**Supplementary Figure 5:** Bioactivity distributions in the independent testing data from ChEMBL-V33 for each protein superfamily. The Y-axis represents the total number of interactions and the X-axis represents pChEMBL values of inactive interactions (range: 5-13).

**Supplementary Table 1:** The number of epochs and time taken for training a separate predictive models for each protein superfamily.

**
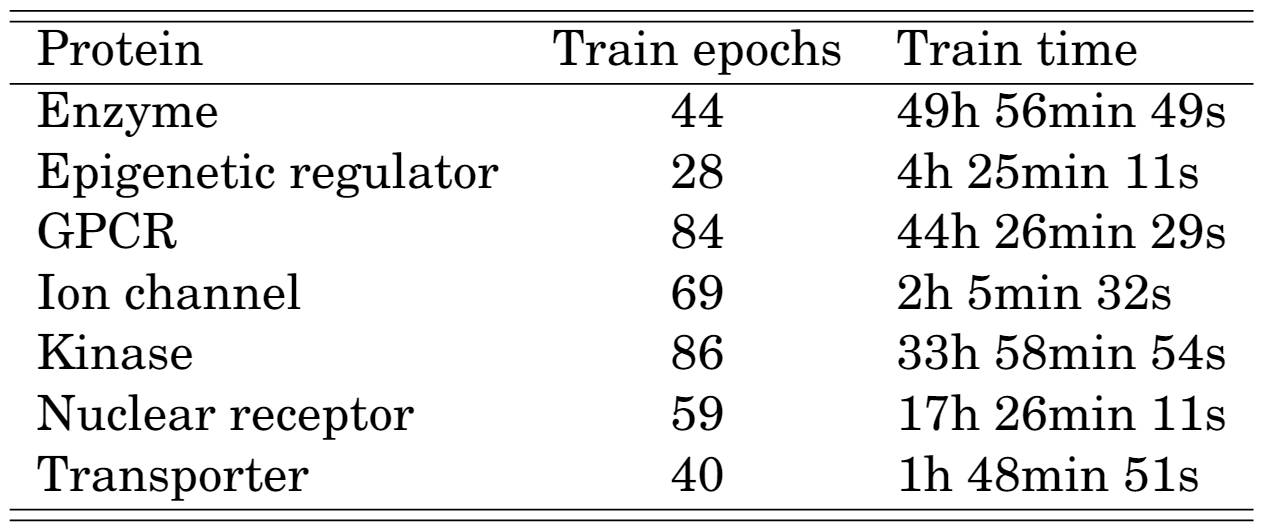
**

**Supplementary Table 2:** The search spaces for tuned hyperparameters and values for constant hyperparameters. We refer to parts of model architecture where compound and protein features are separated and concatenated as "half", and "full", respectively.


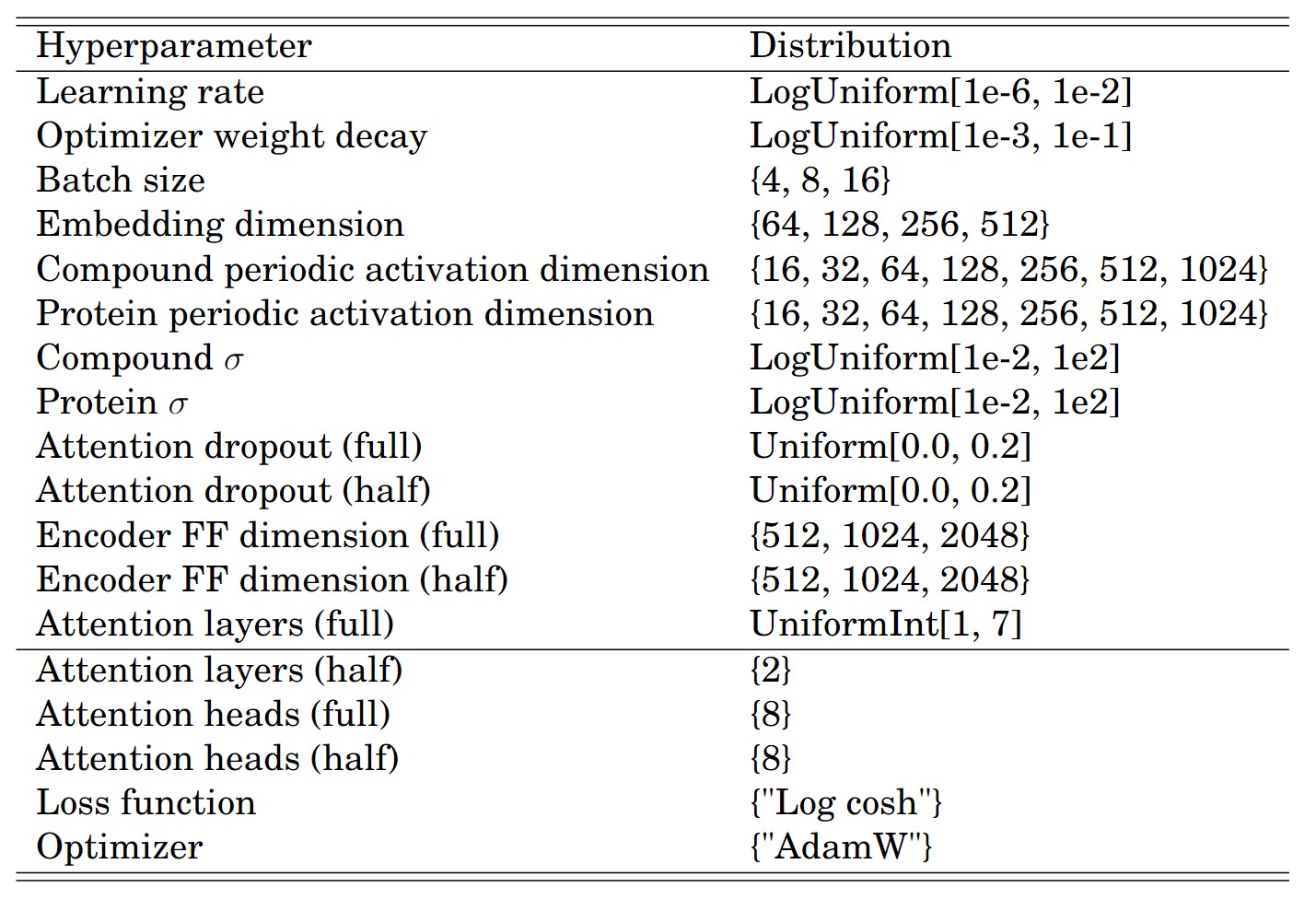


**Supplementary Table 3:** Best hyperparameter values for each protein superfamily after 50 iterations or 144 hours of hyperparameter tuning with HEBO and Hyperband.


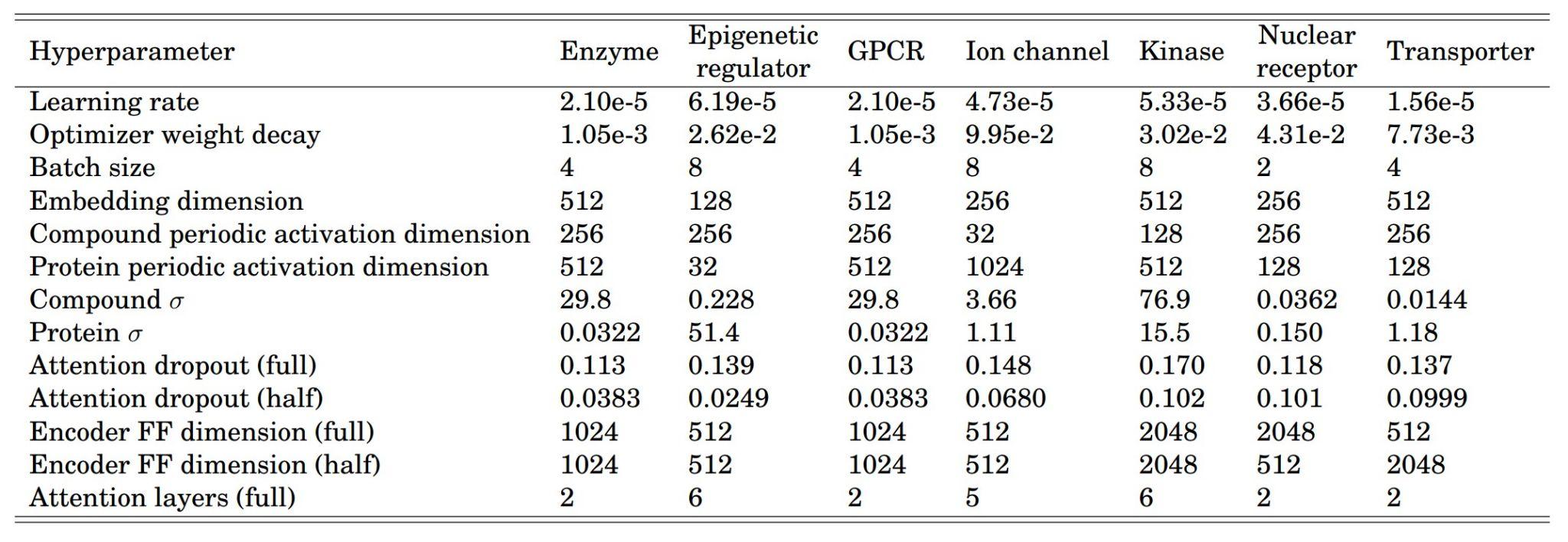


**Supplementary Table 4**: Independent testing results for each protein superfamily in three test scenarios.


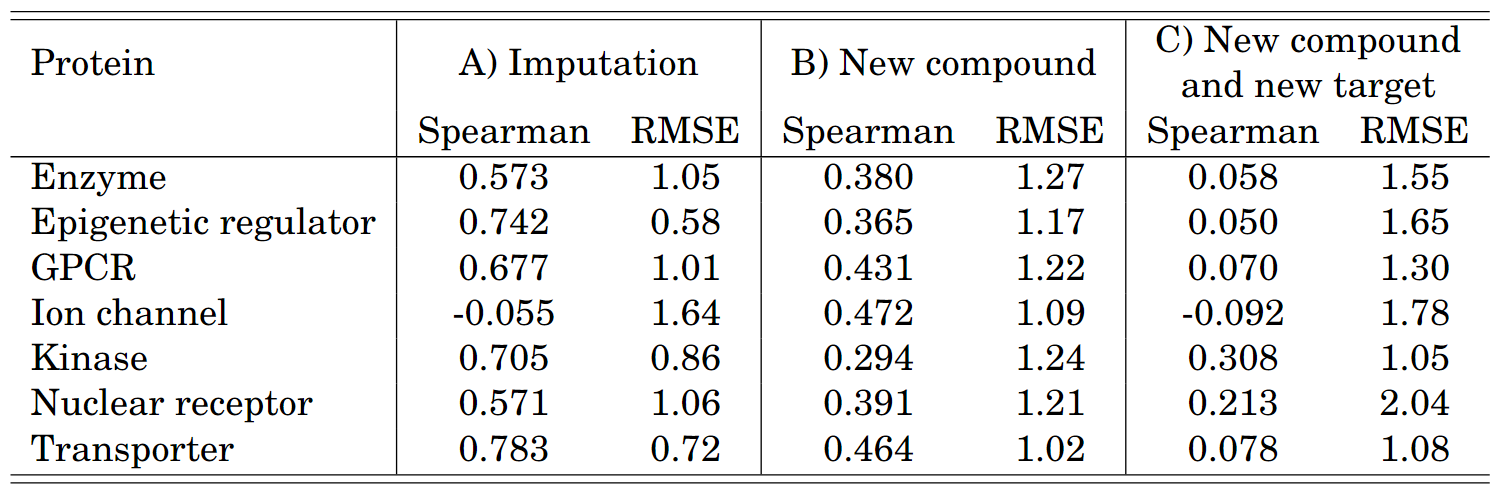


**Supplementary Table 5:** Comparison of our proposed method (MMatt-DTA) with the other models in the Davis dataset. The train-test split is based on the DTITR method. The results are calculated from model predictions on the test split. The best and second-best results are in bold and underlined, respectively.

**
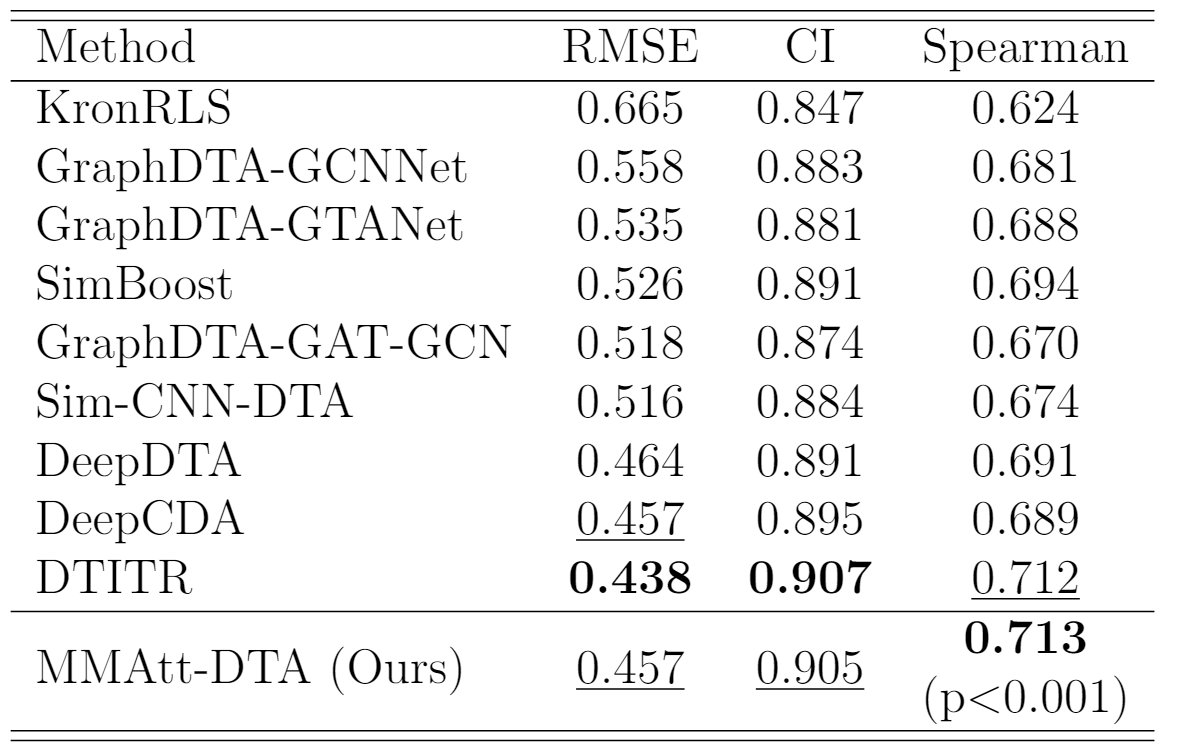
**

**Supplementary Table 6:** Additional comparisons of our proposed method (MMatt-DTA) with five recently published methods in 2023 and 2024. The comparison was performed based on the Davis dataset.


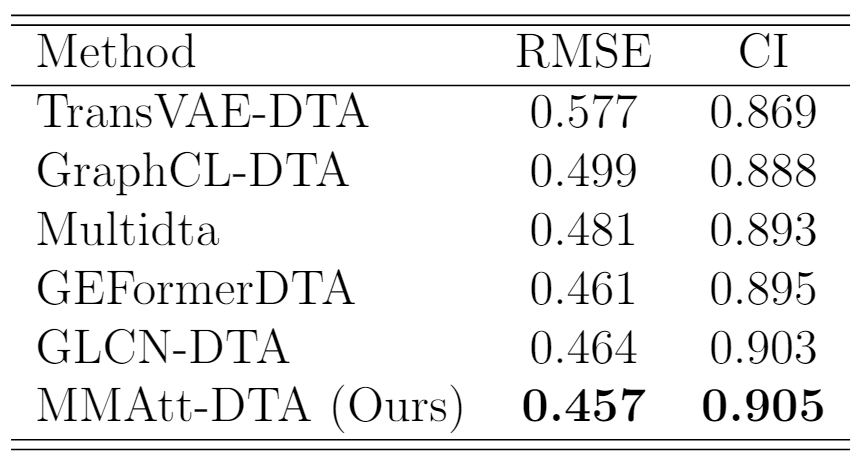


**Supplementary Table 7:** The effect of input feature vector dimension on the performance of the kinase superfamily model. The results improve with higher dimensions. Training the model with 1500-dimensional input and above resulted in GPU memory error.


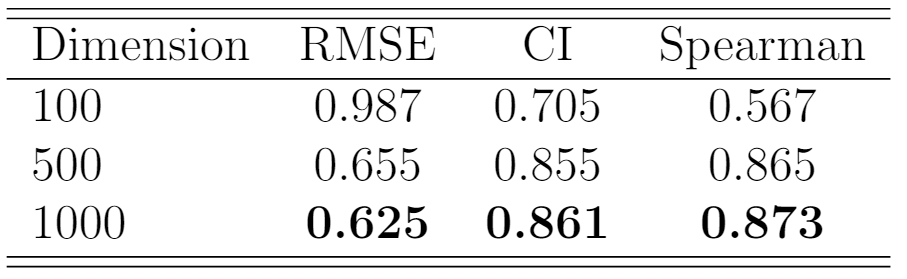


**Supplementary Table 8:** Ablation study to demonstrate the importance of the attention mechanism on the kinase superfamily model. The two versions of the model were trained and tested on the same dataset. Model performance suffers greatly without the attention-based encoders.


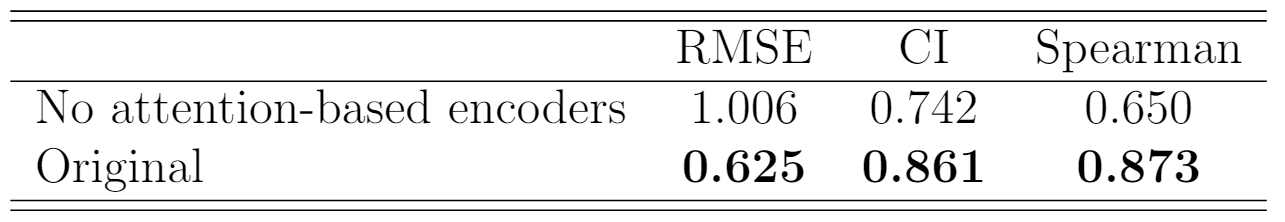

Supplement: btae496_Supplementary_Data [file btae496_supplementary_data.zip › Supplemantary_Figures_Tables.docx]
